# High Diagnostic Accuracy of Nitrite Test Paired with Urine Sediment can Reduce Unnecessary Antibiotic Therapy

**DOI:** 10.2174/1874285801509010150

**Published:** 2015-11-03

**Authors:** Sven A. Ferry, Stig E. Holm, B. Magnus Ferry, Tor J. Monsen

**Affiliations:** 1 Department of Clinical Microbiology, Bacteriology, Umea University, Umea, Sweden; 2 Department of Medical Microbiology and Immunology, University of Gothenburg, Gothenburg, Sweden; 3 Department of Education, Umea University, Umea, Sweden

**Keywords:** Antibiotic resistance, bacteria, bladder incubation time, diagnosis, dipstick test, leukocytes, primary care, urinary tract infection

## Abstract

**Background:**

Urinary tract infections (UTIs) are common bacterial infections dominated by lower UTI in women (LUTIW). Symptoms only are insufficient for diagnosis and accordingly, near patient diagnostic tests confidently confirming significant bacteriuria are desirable. The nitrite test (NIT) has low sensitivity, while bacterial and leukocyte counts disjunctively paired in urine sediment microscopy (SED) have high sensitivity. Similar symptomatic cure rates are found post antibiotic vs. placebo therapy in patients with negative cultures. Consequently, prescription on symptoms only implies unnecessary antibiotic therapy.

**Aims:**

to evaluate the diagnostic outcomes of NIT, SED and NIT disjunctively paired with SED (NIT+SED) vs. urine culture, with special focus on bladder incubation time (BIT), and to assess if NIT+SED can reduce unnecessary antibiotic therapy.

**Methods:**

A diagnostic, primary care, multicentre study including 1070 women with symptoms suggestive of lower UTI.

**Results:**

Significant bacteriuria was found in 77%. The BIT highly influenced the diagnostic outcomes and the optimal duration was ≥4h with sensitivity of 66, 90 and 95% for NIT, SED and NIT+SED, respectively. SED performed only in NIT negative specimens could reduce unnecessary antibiotics by 10% vs. prescription on symptoms only. The number needed to test with SED to reduce one unnecessary antibiotic course was five patients at BIT ≥4h and six patients at ≤3h or overall.

**Conclusion:**

The BIT highly influences the diagnostic outcomes with the highest accuracy of NIT+SED. Diagnosis of LUTIW with NIT+SED can reduce unnecessary antibiotic therapy and subsequently decrease antimicrobial resistance.

**Trial registration:**

The Swedish Medical Product Agency 1995 03 01:151:01783/94.

## INTRODUCTION

 Urinary tract infections (UTIs) are common bacterial infections [1], estimated to 150 million episodes annually worldwide [2], dominated by uncomplicated, community-acquired, lower UTI in women (LUTIW) and often handled in primary care (PC) [3-5]. 

 Patients with LUTIW often want rapid relief of intensive symptoms [6]. However, studies of UTI seldom focus on symptoms, as reported by only 9/464 studies in a meta-analysis [7]. Women with at least one symptom had 50% probability of UTI, while presence of two or more symptoms obtained 65% sensitivity and 69% specificity in a multi-center study [8]. A meta-analysis of symptomatic womenconcluded that clinical findings do not aid in the diagnosis [9], while a review found that individual symptoms only modestly increase the pretest probability of UTI [10]. Moreover, in a multicenter, placebo-controlled, therapy study, the LUTIW project, we found similar mean symptoms scores in women with negative culture as in those with significant bacteriuria (SBU) [6, 11]. Accordingly, symptoms only are insufficient for the diagnosis of UTI.

 The nitrite test (NIT) is the most commonly used near patient diagnostic test with varying diagnostic outcomes [12-14], as 20-85% sensitivity in one meta-analysis [15] *vs*. 38-62% in another [16], which also reported 73-100% specificity depending on different criteria for SBU. 

 UTIs frequently cause inflammation with increasing leukocyturia, often examined by urine microscopy, which however has no uniform methods or interpretive standards accepted and highly variable outcomes [12, 17]. Accordingly, a review of microscopy for bacteria with four different methods reported 61-96% sensitivity and 65-96% specificity [18], and a multicenter study of SED found 47% sensitivity and 81% specificity for bacteria *vs*. 87 and 29% for leucocytes [19]. Though, high-power microscopy of leucocytes in stained SED obtained high inter-observer agreement [20]. A PC study analyzing bacteria disjunctively paired with leucocytes in SED (either or both positive defined as UTI) found 97% sensitivity and 39% specificity [21].

 Previously, we reported similar symptomatic cure rates post pivmecillinam *vs*. placebo therapy in women with negative urine culture, but significantly higher post pivmecillinam in those with SBU [6, 11]. Hence, we concluded that SBU should be confirmed confidently before antibiotics are prescribed. In contrast, current management guidelines in the US [22], Norway [23], Scotland [24] and Sweden [25] recommend antibiotic prescription based on symptoms only. Moreover, empirical treatment is considered most cost-effective, but implies unnecessary antibiotic prescriptions [26] and subsequently increasing antimicrobial resistance [27]. Thus, bacterial antibiotic resistance is an increasing global problem related to the consumption of antimicrobials [28-31] and differing between antibiotics [32]. Also, the simultaneous decline in research of new antimicrobials is now threatening us back to the pre-antibiotic era [33]. However, decreased antibiotic resistance was found following reduction in antimicrobial prescribing by general practices [34]. Accordingly, it is important to reduce unnecessary antibiotic therapy. 

As the general quality of reporting studies of diagnostic accuracy is not optimal [35], the STARD (*Sta*ndards for *R*eporting of *D*iagnostic Accuracy) statement was developed to improve this quality [36]. In the LUTIW project NIT, SED and disjunctive pairing of NIT and SED (NIT+SED) were examined *vs*. urine culture [11]. This diagnostic process is further analyzed as follows:

 The aims of the present study were to evaluate the diagnostic outcomes of NIT, SED and NIT+SED *vs*. urine culture as reference standard, with special focus on the influence of BIT and applying the STARD statement, and to assess if NIT+SED can reduce unnecessary antibiotic therapy in women with symptoms suggestive of lower UTI.

## MATERIALS AND METHODS

### Inclusion

 Women aged 18 years and above with symptoms suggestive of lower UTI were considered for enrolment in the study, which was performed during ordinary office hours at 18 PC centers in northern Sweden between April 1995 and February 1998 [6, 11].The symptoms urgency, dysuria, suprapubic pain or loin pain were registered and graded as none, light, moderate or severe (score 0-3). A total symptoms score of ≥2 was required for inclusion. Oral and written information were given and patients accepting participation gave written consent. 

### Exclusion

 Patients were excluded as earlier reported [6, 11], including pregnancy, antibiotic therapy for UTI within the last month, genital infection, suspected pyelonephritis (temperature of ≥ 38.5^o^C, CRP ≥ 25 mg/L or kidney tenderness by palpation), complicating factors as diabetes or abnormality of the urinary tract, urine incontinence requiring catheter or pads, or previous participation in the study. 

### Study Design and Approvals

 The present study evaluated data from a prospective, consecutive, randomized, double-blind, placebo-controlled, PC, multicentre, therapy study [6, 11]. Age (years), symptoms duration (days), symptoms scores (mean values) and BIT (hours, h) were registered at inclusion. The diagnostic outcomes of NIT, SED and NIT+SED were calculated in relation to urine culture as the reference standard. The STARD statement for reporting diagnostic studies was applied [35, 36]. The study was conducted in accordance with the Swedish Medical Product Agency guidelines and was approved by the Agency 1995-03-01:151:01783/94 as well as by the Ethics Committee of Umea University 1995-03-07 (dnr 93-178, §195/9310, date 930924). 

### Bladder Incubation Time and Urine Sampling

 The longest duration of BIT, or ≥4h if possible, was recommended and most easily achieved in first morning urine samples. Thus, morning visits with such specimens were offered at the PC centers to patients accepting this management. Instructions were given including how to collect an MSU after separating the labia without prewashing the perineum. Urine specimens collected outside the PC centers were instructed to be refrigerated in a clean glass container before transported in a plastic bag containing ice cubes for delivery at the centers. The specimens were separated in two parts, and in the first part NIT and SED were performed immediately.

### Near patient Diagnostic Tests: NIT and SED

 Bacteria can reduce nitrate in the urine to nitrite, as indicated by a pink to red color change of the NIT dipstick. According to the manufacturer (Nitur-test^®^,Boehringer-Mannheim, Mannheim, Germany), the NIT is calibrated for ≥10^5 ^CFU/mL, and the uropathogens need to act in the urine for ≥4h to achieve the highest diagnostic accuracy. Within one minute after the stick was dipped in the urine, any color change was recorded, and at least light pink was defined as positive NIT.

 The SED was performed as previously reported [21]. In summary, a 10-mL tube with conical bottom was filled with urine, centrifuged for five minutes at 3750 rounds/minute (1250 G), followed by careful decanting of the supernatant. One drop of Sternheimer-Malbin wet stain (Sedi-Stain^®^, Becton, Dickinson and Company, Stockholm, Sweden) was added to and mixed with the sediment [37]. Then, a plastic capillary tube (inner diameter 2.5 mm) was dipped in the suspension, and 3µL (range 2-5) was applied to a glass slide before a 18x18 mm cover slip was placed on top [21]. Samples were examined with a light microscope (Zeiss standard model, Carl Zeiss AB, Stockholm, Sweden) having a 40/0.65 achromatic objective and a x 10 wide angle ocular for phase-contrast microscopy yielding a view field with a diameter of 450 µm and a depth of focus of 1.3 µm per high power field ( HPF, magnification x 400). At least five view fields were examined, and the average number of leukocytes and bacteria/HPF were recorded. Leukocytes were counted up to 15/HPF and then approximated as 20, 25, or ≥30/HPF. The bacteria were difficult to count exactly due to their small size and often movements, but were classified as negative (no or a few bacteria), low (10–99), moderate (100-300) or high (innumerable) counts of bacteria/ HPF. The disjunctive pairing of at least a moderate bacterial count and ≥5 leukocytes/HPF was defined as positive SED.

 This SED technique was illustrated in the county management guidelines for UIT in PC [38], which was introduced for laboratory staffs and physicians more than ten years before the start of the LUTIW project. At introduction of the project, all physicians and staff members involved were instructed of the centrifugation and microscopy technique according to a protocol, including a collection of colored sediment images. In addition, magnified sediment samples were shown. At each centre the centrifuge and microscope were calibrated, followed by a supervised practice session. The SED analyses were performed by experienced laboratory technicians at three centers, but by physicians or assistant nurses at the other 15 centers.

### Bacteriological Methods

 The second part of the urine specimen was transported in a 10-mLsterile glass tube chilled at<6^○^C within 24 h to the Laboratory of Clinical Bacteriology, University Hospital of Umea for urine culture [11]. In summary, 10 µL was inoculated on cystine-lactose-electrolyte deficient agar (CLED, Acumedia Manufacturers, Inc. Baltimore, Maryland, USA), and incubated at 35^○^ C for 18-20 h. The uropathogens were quantified and reported in CFU/mL, and identified as previously described. SBU was defined according to guidelines stating cut-off levels of ≥10^3 ^for primary (*E. coli* and *S. saprophyticus*), ≥10^4 ^for secondary and ≥10^5 ^CFU/mL for doubtful uropathogens [12]. In cultures with mixed flora and one predominant species (at least 10-fold higher CFU/mL than that of any other species), the major species were defined as an uropathogen [11]. Non-significant bacteriuria was defined as negative culture. Samples with mixed flora without one predominant species were considered contaminated and defined as negative culture. 

### Statistical Analyses

 The statistical outcomes sensitivity, specificity, predictive values and false results were calculated using the predictive value theory [39]. Efficacy was defined as the percentage correct diagnosis, i.e. 100 minus false (positive and negative) results. Comparison of proportions was done using chi-square test or Fisher’s exact test. P-values <0.05 were considered as statistically significant. The software used for statistical calculation was IBM SPSS 21.0 (IBM Corp., Armonk, New York, USA) and Stat View version 5.0.1 (SAS Institute Inc., Cary, North Carolina, USA).

## RESULTS

**Fig. (1):**
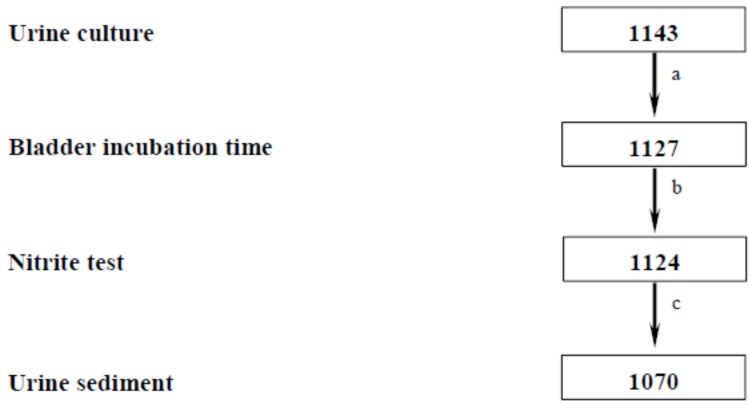
Flow chart of the diagnostic parameters reported in women with symptoms suggestive of lower UTI resulting in patients included in the diagnostic study (n). ^a^ Bladder incubation time missed in 16 patients; ^b^ Nitrite test missed in three more patients; ^c^ Urine sediment missed in 54 further patients.

 In total, 1143 women with urine culture performed were eligible for enrolment in the present study (Fig. **1**.) Of those, 73 patients (6%) were excluded due to missing BIT in 16 patients, NIT in three more and SED in another 54. The remaining 1070 patients included had a mean age of 43 years (±18), median symptom duration of four days and mean symptoms score of 5.3 (range 2-12). The symptoms reported were 96% urgency, 88% dysuria, 60% suprapubic pain and 40% loin pain. The excluded patients had similar characteristics, symptoms and culture results as those included (data not shown).

 SBU was found in 77% of the included patients (827/1070), of which *E. coli* dominated in 81% (Table **1**). 

 Mixed bacterial flora was found in 13%, of which 7% with one predominant species were reported as a uropathogen and 6% were reported as culture negative. The mean BIT was 4.2h (±2.5) and 56% of the patients achieved a BIT of ≥4h (Table **2**). The bacterial counts in SED and urine cultures were highly influenced by the duration of BIT (p<0.001), especially for *E. coli* (Table **1**). This was illustrated by the high bacterial counts of ≥10^5 ^CFU/mL in 94% at BIT ≥4h *vs*. 71% at BIT ≤3h**. **Since no further increase of bacterial counts was observed for BITs >4h, the BIT ≥4h was found to be the optimal duration and thus used as cut-off level in the further analyses. Negative culture was found in 243 patients (23%) without significant influence by the BIT (25% at BIT ≤3h *vs*. 21% at ≥4h, p=0.123). 

 The diagnostic outcomes of NIT were highly influenced by the BIT. The sensitivity and efficacy increased from 30 and 47% for BIT ≤3h to 66 and 72% for ≥4h (p<0.001, Table **3**). However, the BIT had lower impact on the specificity and positive predictive value, which on average were found in 95 and 97%. *E. coli* had the highest sensitivity increasing from 60 to 63 and 67% for bacterial counts of 10^3^, 10^4^ and ≥10^5^ CFU/mL compared to 49, 52 and 57% in all culture positive specimens**,** respectively.

The BIT had lower impact on the outcomes of SED, which were similar and consistent at all centers during the study period (data not shown). However, the bacterial counts highly increased by the duration of BIT (p<0.001) but the leukocyte counts were similar. Accordingly, the sensitivity of SED was 88% fort BIT ≤3h and 92% for BIT ≥4h (p=0.079, Table **3**). The influence of BIT on the other diagnostic outcomes was low, with averages of 46% specificity and 85% positive predictive value. Thus, the diagnostic efficacy was overall higher for SED compared to NIT (80 *vs*. 61%, p<0.001).

 Since the BIT had also high influence on the diagnostic outcomes of NIT+SED, the sensitivity increased from 89% for BIT ≤3h to 95% for BIT ≥4h (p<0.01, Table **3**). Also**, **the efficacy increased from 79 to 84%, respectively (p<0.05). In contrast, the false negative results decreased from 8% for BIT ≤3h to 4 % for ≥4h (p<0.01). However, the BIT had low impact on specificity and positive predictive value, which on average were found in 44 and 85%. Accordingly, NIT+SED had the highest diagnostic accuracy with the highest efficacy overall for BIT ≥4h (84%), which was significantly higher compared to NIT (72%, p<0.001) and tended to be higher than for SED (81%, p=0.585). 

**Fig. (2):**
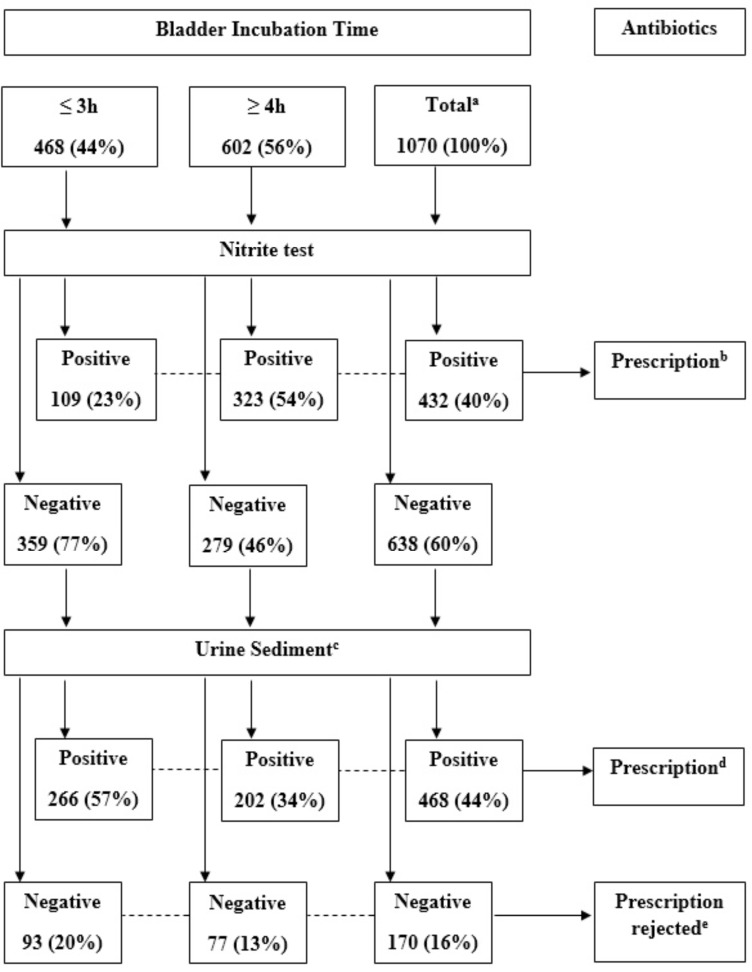
Bladder incubation time, nitrite test and urine sediment vs. urine culture in diagnosing women with symptoms suggestive of lower UTI resulting in prescription of antibiotics or prescription rejected. ^a^ Overall, irrespective of bladder incubation time (BIT); ^b^ Incorrect prescription in three patients at BIT ≤3h (1%), eight at BIT ≥4h (1%) and 11 overall (1%); ^c^ Urine sediment, see Methods; ^d^ Incorrect prescription in 59 patients at BIT ≤3h (13%), 66 at BIT ≥4h (11%) and 125 in total (12%); ^e^ Prescription incorrectly rejected in 38 patients at BIT ≤3h (8%), 25 patients at BIT ≥4h (4%) and 63 patients overall (6%).

Further, we evaluated the diagnostic outcomes in a management model when SED was performed only in NIT negative specimens (Fig. **2**), which occurred in 77% at BIT ≤3h, in 46% at BIT ≥4h and on average in 60% (Table 2). With this management model patients would have been prescribed antibiotics overall to 80% (23 + 57%, Fig. 2) and unnecessarily to 13% (false positive, Table 3) at BIT ≤3h *vs*.88 and 12% at BIT ≥4h (Fig 2. and Table 3), and overall to 84 and 13%, respectively. Accordingly, compared to antimicrobial treatment based on symptoms only, application of NIT+SED could reduce unnecessary antibiotic prescriptions by 12% (25-13%, Tables 1 and 3) at BIT ≤3h, by 9% (21-12%) at BIT ≥4h and on average by10% (23-13%). Thus, the number needed to test with SED, in order to reduce one unnecessary antibiotic course, were five patients at BIT ≥4h (1/0.09 x 0.46 = 5.11) and six patients at BIT ≤3h (1/0.12 x 0.77 = 6.42) as well as six patients overall (1/0.10 x 0.60 = 6.00). 

## DISCUSSION

 To our knowledge, the present study is the first comprehensive multicenter study of uncomplicated LUTIW in PC evaluating the diagnostic outcomes of NIT, SED and NIT+SED with focus on the influence of BIT. Since information about the importance of long duration of BIT was emphasized, just more than half of the patients achieved a BIT of ≥4h, which highly increased the bacterial counts in urine culture as in SED. Accordingly, SBU was obtained in 77% of the patients, which frequency is higher as compared to most studies in literature [15, 16, 40-43]. As the BIT also had high impact on NIT, the sensitivity of NIT was higher than in literature. Since NIT obtained very high specificity and positive predictive value overall, a positive NIT confidently confirmed diagnosis of UTI, which is in accordance with literature. SED had higher diagnostic accuracy than NIT but similar to that reported in a previous PC study [21]. The BIT also highly influenced outcomes of NIT+SED, which had the highest diagnostic efficacy for BIT ≥4h. 

 We evaluated the management model with SED performed only in NIT negative specimens, which overall concerned 60% of the patients. This management resulted in antibiotic prescription overall to 84% and unnecessarily to 13%. Moreover, this model was effective for reducing unnecessary antimicrobial therapy, as the number needed to test with SED, in order to prevent one unnecessary course of antibiotics, were only five patients at BIT ≥4h and six patients at BIT ≤3h as well as six patients overall.

 In most guidelines for the evaluation of new antimicrobial treatment of UTI, neither BIT nor sampling technique is mentioned [22-24, 45], which also concern most published diagnostic studies of UTI [15, 16, 40, 41]. The Swedish diagnostic guidelines for LUTIW applied in the present study [12] have cut-off levels for SBU varying from ≥10^3 ^to ≥10^5 ^CFU/mL for different uropathogens. These guidelines were revised in 2000 with preserved levels for SBU [42] and then also approved in Europe [43]. The American diagnostic guidelines for UTI are differing from the other guidelines, with criterion for SBU of ≥10^3 ^CFU/mL for all uropathogens in LUTIW [44]. However, if the American guidelines had been implemented in the present study, this would only have had minor influence on the diagnostic outcomes. Accordingly, we consider that the applied criteria for SBU are valid for the diagnosis of LUTIW.

 The longest possible BIT is recommended in diagnostic guidelines for UTI [12, 42-45], and ≥4h is recommended by the manufacturer for obtaining the highest diagnostic accuracy. We found that BIT highly influenced most diagnostic outcomes, but BIT >4h did not further improve the diagnostic accuracy. Thus, we conclude that the optimal duration of BIT is ≥4h. 

 In the present study MSU specimens were collected after separating the labia without prewashing the perineum. However, the clean-catch sampling technique with separating the labia and cleansing the perineum before collecting a MSU specimen has been recommended for decades [13]. Though, that procedure is difficult both to understand and perform adequately, and its clinical documentation is sparse [46]. Similar distribution of uropathogens and contamination rates were found in home-voided specimens without prewashing as in clean-catch specimens voided at PC centers [47]. Also, similar findings were obtained in MSU specimens collected at PC centers irrespective of clean-catch or not [48-51]. Consequently, the sampling technique for collecting MSU specimens applied in the present study is valid. 

 Both internal and external quality controls are recommended in order to achieve high validity and reliability of near patient diagnostic tests [35, 36]. The local guidelines for the management of UTI in PC including the SED method were introduced for staffs and physicians in PC and repeatedly trained before and after the LUTIW project. This probably increased the quality of management of UTI at the centers in the present study**. **Hence, we recommend PC centers to participate in education and training of management guidelines for UTI.

 Scottish guidelines discourage from use of urine microscopy due to requirements of maintenance of equipment and training of the staff [24]. However, 15 centers with SED performed by physicians or assistant nurses, had similar outcomes of SED as the three centers with experienced laboratory technicians, and the SED data were consistent during the study period supporting the reliability of the method. Furthermore , SED is fast to perform and the material costs are about the same as for urine dipsticks with multiple tests**. **Since SED is also valid and easy to learn, we recommend our proposed diagnostic model of SED for the application in clinical practice.

There are few published studies of UTI performing NIT and SED for diagnostics of UTI. A multicenter study of NIT and high-power microscopy of leukocytes in SED reported 62% sensitivity and 89% specificity for NIT as 84 and 35% for leukocytes [52]. Disjunctive pairing of NIT and leukocytes obtained the highest diagnostic accuracy with 93% sensitivity and 17% specificity. Thus, the sensitivity was similar, but the specificity was lower, compared to NIT+SED in the present study. 

 The urinary leukocytes are currently often examined in clinical practice by the leukocyte esterase test (LE). In one meta-analysis of symptomatic patients the sensitivity range was 40-100% [15] *vs*. 60-98% in a second meta-analysis [16], also reporting 32-68% specificity. The true and false positive rates for the tests were interdependent and remarkably heterogeneous [15]. Outcomes of combined dipsticks with NIT and LE disjunctively paired (NIT+LE) had the highest diagnostic accuracy with 61-93% sensitivity and 63-78% specificity [16]. However, negative NIT+ LE did not rule out infection in a multicenter study of symptomatic women, as SBU was found in 50% of these specimens [14]. Accordingly, combinations of these tests are unreliable for the diagnosis of UTI. 

 In two multicenter studies of symptomatic women, SBU was found in 63 and 66% [8, 53]. A diagnostic model based on nitrite and/or both leukocytes and erythrocytes defined as confirmed UTI, reported 77% sensitivity and 70% specificity in the first study *vs*. 75 and 60% in the second, respectively. Thus, the diagnostic accuracy for combination of those dipstick tests was overall lower *vs*. NIT+SED in the present study. 

 Two other multicenter studies of suspected LUTIW applied different criteria for SBU, which were reported in 53 and 63% [54, 55]. Patients with urgency or dysuria were offered antimicrobial treatment by telephone without urinalysis. Antibiotics were prescribed overall to 81% and unnecessarily to 40% of the patients in the first study *vs*. 89 and 29% in the second, respectively. A management model based on the factors dysuria, nitrite and leukocytes on dipsticks, defined the presence of at least two factors as confirmed UTI diagnosis and recommended antimicrobial prescription. The presence of one of the factors was considered as unreliable and proposed to await outcome of urine culture for confident diagnosis. If that management was applied, antibiotic prescriptions would have decreased overall to 60% and unnecessarily to 28% in the first study *vs*. 68 and 17% in the second, respectively. Hence, lower diagnostic accuracy and higher frequency of unnecessary antibiotic prescriptions were obtained *vs*. management with NIT+SED in the present study.

 In the present study, the sensitivity of NIT+ SED was higher than for most studies of NIT+LE according to literature [15, 16, 40, 41], presumably due to the disjunctive pairing of bacteria and leucocytes in SED. NIT+SED had the highest diagnostic accuracy for BIT ≥4h, with a very high sensitivity and a low proportion of false negative results, resulting in few incorrectly rejected antibiotic prescriptions. Moreover, most patients had decreasing symptoms by placebo therapy [6] and only one of those 288 patients developed pyelonephritis [11]. Accordingly, we consider that these minor risks for complications achieved by NIT+SED are satisfactory and up to now**,** this is the most accurate model for near patient diagnostics of LUITW.

 We found that our proposed management model with NIT+SED applied in patients with symptoms suggestive of LUTIW, can reduce unnecessary antibiotic prescriptions by an average of 10 % *vs*. prescription based on symptoms only, which subsequently can decrease antimicrobial resistance. If this management was implemented in clinical practice worldwide, this reduction could be estimated to 15 million episodes annually [2]. However, the abuse of unnecessary antimicrobials is probably much more extensive, as considerably lower proportions of SBU are found in clinical practice in patients with symptoms suggestive of lower UTI as compared to the present study. This is previously reported [8, 53-55] and also illustrated by a multicenter study with SBU found in only 21% of patients with suspected UTI [56]. Consequently, even larger reduction of unnecessary antimicrobial consumption and decreased antibiotic resistance can be expected than those indicated in the present study.

 The strengths of the presents study are firstly, the comprehensiveness with high frequency of SBU and low proportion of contaminated cultures. Secondly, the optimal BIT of ≥4h was obtained in many patients with high influence on the diagnostic process. Thirdly, the STARD statement for accurate reporting of diagnostic studies was applied, whereby methods and results were thoroughly described in both the present study and literature. We presume that this enable the readers to assess the potential for bias in the present study and to evaluate the generalisability of the results. 

 The limitations of the study are firstly, that the LUTIW project was performed more than ten years ago. However, the criteria for SBU and the distribution of uropathogens causing uncomplicated LUTIW have remained similar for decades and accordingly, the results of the present study are still valid. Secondly, the study was performed at ordinary office hours. Though, if patients also had been included at out of office hours**, **this probably would have resulted in shorter BITs and lower diagnostic accuracy. Thirdly, after planning of the study, dipsticks with multiple tests including LE for urinalysis were introduced in clinical practice, which however, have lower diagnostic accuracy *vs*. NIT+SED in the present study.

In order to obtain high diagnostic accuracy in patients with symptoms suggestive of UTI, pre analytical procedures have to be highlighted. These procedures concern BIT, sampling technique, storage, transport and preparation of the specimens for urinalysis, as well as careful and well-standardized performance of the diagnostic methods [57], which were given high attention in the present study. The applied MSU sampling technique was easy for the patients to understand and perform. Many patients accepted morning visits and first morning specimens**, **which extent we unfortunately did not register. However**,** our experience supports that this is easy to implement, if patients are informed by the staffs and physicians about the importance of BIT ≥4h. To summarize, we consider that our proposed management model with NIT+SED is valid and suitable for patients, staffs and physicians to implement in clinical practice, and also for researchers to implement in the management guidelines for UTI. 

 As studies of urine microscopy are rather sparsely published, further diagnostic multicenter studies of patients with symptoms suggestive of lower UTI in PC from different countries evaluating SED are desirable, but also studies combining SED with multiple dipstick tests. Hopefully, such studies will verify the results from the present study and also further improve the management of UTI in clinical practice.

## CONCLUSION

NIT+SED have the highest diagnostic accuracy of near patient diagnostic tests for LUTIW.

 The BIT highly influences the diagnostic outcomes of LUTIW with ≥4h as optimal duration.

Implementation of NIT+SED can reduce unnecessary antibiotic prescriptions in LUTIW and subsequently decrease antimicrobial resistance.

 The number needed to test with SED in NIT negative specimens, in order to reduce one unnecessary course with antibiotics, is only five patients at BIT ≥ 4h and six patients at BIT ≤3h as well as six patients overall.

## Figures and Tables

**Table 1:** Distribution of bacterial species and bacterial counts in urine cultures in relation to bladder incubation time in women with symptoms suggestive of lower UTI.

Urine culture and bacterial species	BC^a^	Bladder incubation time			
		≤ 3h	≥ 4h	Total	
		n	n	n	%
*E.coli*٭٭٭	10^3^	27	10	37	6
	10^4^	55	12	67	10
	≥10^5^	202	361	563	84
	Total	284	383	667	62
Other G-neg^c^ spp^b^	10^4^	2	0	2	4
	≥10^5^	12	42	54	96
	Total	14	42	56	5
*S. saprophyticus *	10^4^	3	5	8	12
	≥10^5^	31	30	61	88
	Total	34	35	69	6
Other G-pos^d^ spp^b^	10^4^	5	3	8	23
	≥10^5^	14	13	27	77
	Total	19	16	35	3
Negative culture^e^	0^e^	117^f^	126^g^	243	23
All cultures٭٭٭	0^e^	117	126	243	23
	10^3^	27	10	37	3
	10^4^	65	20	85	8
	≥10^5^	259	446	705	66
Total	n	468	602	1070	
	%	44	56	100	100

^a^Bacterial counts in colony forming units/mL; ^b^ Species; ^c^ Other Gram-negative spp: 28 *Klebsiella* spp, 12 *Citrobacter*, 12 *Enterobacte*r, 6 *Proteus* spp, 2 *Pseudomonas* spp; ^d^ Other Gram-positive spp*: 21 Enterococcus spp, 7 S. auerus, 5 Coagulase-negative staphylococci *other than *S.saprophyticus*, 5 *Group B streptococci*; ^e ^Negative culture, see Methods; ^f ^25% of all negative cultures; ^g^ 21% of all negative cultures; ٭٭٭p<0.001: Statistical differences in bacterial counts between bladder incubation time ≤ 3h *vs*. ≥ 4h.

**Table 2:** Outcomes of nitrite test, urine sediment and urine culture in relation to bladder incubation time in women with symptoms suggestive of lower UTI (n).

BIT^a^	Nitrite test^b^	Urine sediment^c^	Urine culture^d^		
			Negative	Positive	Total
≤ 3h	Negative^e^	Negative	55	38	93
		Positive	59	207	266
	Positive	Negative	2	5	7
		Positive	1	101	102
	All		117	351	468
≥ 4h	Negative^f^	Negative	52	25	77
		Positive	66	136	202
	Positive	Negative	2	15	17
		Positive	6	300	306
	All		126	476	602
Total	Negative^g^	Negative	107	63	170
		Positive	125	343	468
	Positive	Negative	4	20	24
		Positive	7	401	408
	All	** **	243	827	1070

^a^ BIT, bladder incubation time (h); ^b^ Positive Nitrite test, see Methods; ^c^ Positive Urine sediment, see Methods; ^d^ Positive Urine culture, see Methods; ^e ^77% of all patients at BIT ≤ 3h; ^f^ 46% of all patients at BIT ≥ 4h; ^g^ 60% of all patients overall irrespective of BIT.

**Table 3:** Statistical outcomes of nitrite test, urine sediment and their disjunctive pairing in relation to bladder incubation time in women with symptoms suggestive of lower UTI (%).

BIT^a^	Diagnostic tests	Sensitivity	Specificity	Predictive value		False results		Efficacy^b^
				Positive	Negative	Positive	Negative	
≤ 3h	NIT^c^	30	97	97	32	1	52***	47
	SED^d^	88	49	84	57	13	9	78
	NIT+SED^e^	89	47	83	59	13	8**	79
≥ 4h	NIT^c^	66***	94	98	42**	1	27	72***
	SED^d^	92	43	86	57	12	7	81
	NIT+SED^e^	95**	41	86	68	12	4	84*
Total	NIT^c^	51	95	97	36	1	38	61
	SED^d^	90	46	85	57	12	8	80
	NIT+SED^e^	92	44	85	63	13	6	81

^a^ BIT,^, ^bladder incubation time (h); ^b ^Correct diagnosis (100 minus false positive and false negative results); ^c ^NIT, nitrite test, see Methods; ^d ^SED, urine sediment, see Methods; ^e^ NIT+SED, disjunctive pairing of NIT and SED (either and/or both positive defined as UTI); *p<0.05; **p<0.01; ***p<0.001: Statistical differences in proportions between BIT ≤3h *vs*. ≥4h.
